# Affective memories of school physical education, life-phase timing of formative experiences, and adult sport and exercise participation: a quota-representative study in Germany

**DOI:** 10.1186/s12889-026-28587-4

**Published:** 2026-07-17

**Authors:** Ralf Brand, Reinhold Kliegl

**Affiliations:** University of Potsdam, Potsdam, Germany

**Keywords:** Motivation, Affect, Development, School

## Abstract

**Background:**

School physical education (PE) is one of the few contexts in which nearly all individuals encounter structured movement. We explored whether adults’ PE memories relate to sport and exercise participation across behavioral dimensions such as engagement, time investment, and participation style.

**Methods:**

Using a quota-representative sample of adults living in Germany (*N* = 2,201), we examined whether the affective valence of adults’ memories of PE (PEMV) and the timing of self-reported formative sport and exercise experiences (FSEE) were associated with adult sport and exercise behavior. Analyses distinguished between three behavioral layers: whether individuals participated in sport and exercise, how much time active adults spent on sport and exercise, and how participation was organized across the week. A hurdle model was used for engagement versus non-engagement, and linear mixed models were estimated for engagement volume and participation style among active adults.

**Results:**

PEMV was most strongly associated with whether adults reported participating in sport and exercise, indicating a gatekeeping pattern. Most respondents remembered their formative experiences occurring in childhood or adolescence, and within these groups, more positive PE memories were associated with lower odds of non-engagement. The smaller group of respondents identifying adulthood as their formative period showed a decoupling of PE memories and sport and exercise participation. Among active adults, associations between PEMV and engagement volume depended on FSEE. Participation style was unrelated to PEMV and FSEE, but varied systematically with age, with younger adults tending toward fewer longer sessions and older adults toward more frequent shorter sessions.

**Conclusions:**

Our findings suggest that school PE may function as a population-level motivational gatekeeper for later sport and exercise participation. At the same time, the apparent decoupling of PEMV and participation in adults identifying adulthood as their formative period suggests that negative PE memories need not remain associated with lifelong disengagement. Later opportunities to choose forms of movement that better match individual preferences may provide second entry points into engagement. Differences in how sport and exercise are organized across adulthood suggest that promotion efforts may benefit from recognizing that sustained participation can take different forms in everyday life.

Regular engagement in physical activity is one of the most robust behavioral predictors of health and longevity across adulthood [1, 2]. Higher activity levels are consistently associated with lower cardiovascular risk [3], reduced all-cause mortality [4], and broad benefits across the adult lifespan [5, 6]. Yet despite this evidence, a substantial proportion of adults remain insufficiently active [7]. The central question is therefore not whether physical activity matters for health, but why it becomes a stable part of some adult lives while remaining largely absent from others.

One influential line of research suggests that the roots of adult engagement lie in earlier life experiences, particularly during childhood and adolescence. Longitudinal studies show that activity patterns track across the life course [8, 9]. However, what carries forward is not behavior alone. Earlier movement experiences may also leave enduring psychological traces. Such experiences may be especially influential because they help establish, from an early age, how movement is perceived, remembered, and affectively evaluated. As a result, sport and exercise may later be perceived as an attractive or aversive behavioral option.

Physical education (PE) occupies a distinctive position in this regard. In many education systems, PE is mandatory and therefore represents a near-universal exposure to organized sport- and exercise-related activities. School environments, including PE, have also been linked to long-term health outcomes and later physical activity behavior [10, 11]. For many individuals, PE is one of the earliest and most sustained contexts in which movement is practiced, experienced, and evaluated. It may therefore leave not only behavioral traces, but also affectively colored memories that shape later orientations toward sport and exercise.

Against this background, initial studies have explored whether adults’ recollections of school PE experiences relate to physical activity behavior later in life. In particular, adults’ affective evaluations of PE, reflecting how positively or negatively school PE is remembered, have typically been examined as bivariate correlations with current activity-related attitudes, intentions, and behavior [12–14]. Although still limited and often interpreted in ways that come close to suggesting a causal link between past experiences and present behavior, this emerging line of research suggests that affectively remembered early movement experiences may have lasting motivational relevance. More broadly, it resonates with a growing emphasis in exercise psychology on affective processes as important determinants of motivation and behavioral regulation [15–18].

In the present study, we focus specifically on participation in sport and exercise. Compared with many everyday forms of movement, sport and exercise often involve higher-intensity and deliberately organized activity. They therefore represent a comparatively time-efficient way of accumulating health-relevant physical activity [19–21]. This focus also reflects the context of school PE, where movement experiences are typically taught and practiced through sport and exercise.

Interpreting memories of earlier experiences such as school PE rests on the premise that behavior is guided not by past events themselves, but by how those experiences are mentally represented in the present [22]. Research on autobiographical memory shows that recollections are reconstructive rather than literal records of the past: such memories are shaped by current perspectives, affective evaluations, and narrative interpretations of one’s life history [23–25].

Although such recollections do not reproduce past events with precision, they form psychologically meaningful representations that contribute to identity, attitudes, and behavioral preferences [26, 27]. Remembered experiences therefore become part of the motivational architecture through which individuals interpret their relationship with sport and exercise [28]. From this perspective, understanding remembered experiences may be relevant both for explaining how active lifestyles develop and for identifying factors that may facilitate later engagement among adults who are currently inactive.

To examine how the affective tone of remembered PE experiences relates to adult engagement, we distinguish between three behavioral dimensions of sport and exercise participation: current participation status (active vs. inactive), engagement volume among those who are active, and the temporal organization of activity across the week [29–31].

From a life-course perspective, these dimensions may follow different explanatory logics. Biographical experiences and the ways in which they are remembered, may be especially relevant for whether sport and exercise become part of adult behavioral repertoires at all, whereas the temporal organization of participation may depend more strongly on current life circumstances and constraints that change systematically across adulthood [32]. In addition, formative sport and exercise experiences may occur at different life phases [33], meaning that, for some individuals, decisive experiences are located in childhood or adolescence, whereas others identify them later in adulthood. This timing may shape how sport and exercise are taken up, maintained, and organized in adult life.

In a quota-representative sample of adults in Germany, we explored how the affective valence of PE memories and the timing of formative sport and exercise experiences relate to adult sport and exercise behavior. Rather than treating engagement as a single outcome, we examined its behavioral components separately. This allowed us to test whether remembered PE experiences are linked primarily to the threshold between engagement and non-engagement, or whether their relevance extends beyond this threshold to the amount and organization of activity in adult life. More generally, the study may help clarify whether early experiences with sport and exercise leave motivationally favorable traces in memory that contribute to sustained engagement later in life.

## Methods

The study was conducted in accordance with the principles of the Declaration of Helsinki. Data were collected using Bilendi, a professional panel provider certified according to ISO 20,252 standards for market, opinion, and social research. Respondents who did not complete the questionnaire were excluded during data collection (*n* = 46; 1% of survey starts). Among completed questionnaires, the mean completion time was 112 s (*SD* = 50), consistent with the expected duration of the survey and without indications of systematic response quality problems. Before participating, respondents were informed that participation was voluntary, provided informed consent, and could discontinue the survey at any time.

### Sampling design and data collection

We conducted a quota-representative online survey of adults living in Germany. The questionnaire was administered using the SoSci Survey platform. Data were collected between 11 and 20 November 2025.

Quota specifications were derived from official population statistics of the Federal Statistical Office (Destatis) to align the sample with the German population structure in terms of age, sex, education, and region of residence. Eight hard quotas were applied to reproduce the joint population distribution of age and sex as of 31 December 2024. Regional representation across all 16 federal states was matched to official population shares and monitored during fieldwork using invitation caps [34]. Educational attainment was classified according to the International Standard Classification of Education (ISCED) [35] and matched to the national distribution of formal educational attainment based on data from the German Microcensus [36].

The target sample size of approximately 2,000 respondents was determined a priori based on a combination of feasibility constraints and the goal of obtaining stable estimates across demographic quota groups. Given the quota-based sampling design and planned analyses, this sample size was considered sufficient to address the study objectives.

### Participants

A total of 2,201 adults aged 18–74 years (*M* = 47.1, *SD* = 15.8) completed the survey. Recruitment followed the predefined quota specifications for age, sex, educational attainment, and region of residence in Germany (Table 1). As intended by the quota design, women and men were represented in nearly equal proportions across age groups. Participants covered the full adult age range, varied widely in educational attainment, and were recruited from all 16 federal states.

**Table 1:** Sociodemographic characteristics of the full sample and analytical subsample

	All participants (*N* = 2,201)	Analytical subsample (*n* = 1,917)
	*N* (%)	*N* (%)
Age (years) and sex		
18–29		
Female	216 (49%)	194 (51%)
Male	225 (51%)	188 (49%)
30–44		
Female	283 (49%)	240 (49%)
Male	289 (51%)	247 (51%)
45–64		
Female	395 (50%)	344 (49%)
Male	397 (50%)	355 (51%)
65–74		
Female	208 (53%)	177 (51%)
Male	188 (47%)	172 (49%)
Total		
Female	1102 (50%)	955 (50%)
Male	1099 (50%)	962 (50%)
Education		
Low (ISCED levels 0–2)	641 (29%)	538 (28%)
Mid (ISCED levels 3–4)	1189 (54%)	1045 (55%)
High (ISCED levels 5–8)	371 (17%)	334 (17%)
Region		
Baden-Württemberg (BW)	292 (13%)	254 (13%)
Bavaria (BY)	349 (16%)	319 (17%)
Berlin (BE)	98 (4%)	89 (5%)
Brandenburg (BB)	68 (3%)	64 (3%)
Bremen (HB)	20 (1%)	16 (1%)
Hamburg (HH)	50 (2%)	40 (2%)
Hesse (HE)	167 (8%)	135 (7%)
Mecklenburg-Western Pomerania (MV)	43 (2%)	39 (2%)
Lower Saxony (NI)	211 (10%)	178 (9%)
North Rhine–Westphalia (NW)	470 (21%)	410 (21%)
Rhineland-Palatinate (RP)	110 (5%)	94 (5%)
Saarland (SL)	27 (1%)	25 (1%)
Saxony (SN)	106 (5%)	91 (5%)
Saxony-Anhalt (ST)	58 (3%)	47 (2%)
Schleswig-Holstein (SH)	77 (3%)	69 (4%)
Thuringia (TH)	55 (2%)	47 (2%)

Educational categories follow the International Standard Classification of Education (ISCED), a UNESCO framework for comparing educational levels across countries (https://www.uis.unesco.org/en/methods-and-tools/isced). ISCED 0–2 = no or lower secondary qualification; ISCED 3–4 = upper secondary or vocational qualification; ISCED 5–8 = tertiary education (e.g., bachelor’s, master’s, doctoral degrees)

A subset of 284 participants did not provide PEMV data because they indicated that they could not remember whether they had experienced PE as pleasant or unpleasant (*n* = 177), had been exempted from PE at school (*n* = 75), or had not participated in PE (*n* = 32). These respondents were not included in analyses involving PEMV, resulting in an analytical subsample of 1,917 participants (*M*_age_ = 47.3, *SD*_age_ = 15.8). Descriptive characteristics of both the full sample and the analytical subsample are presented in Table 1. Apart from restricting PEMV analyses to respondents providing analyzable PE memory data, no additional missing data occurred because responses to all variables included in the statistical models were mandatory, while predefined response categories, where available, were retained as valid analytical responses.

Participants not contributing PEMV data differed only marginally from those retained with respect to age, education, sex, and region of residence, with missing PEMV being somewhat more common among older participants and those with lower educational attainment. However, these differences were negligible across all quota variables (maximum absolute proportion difference = 0.011; maximum |Cohen’s *h*| = 0.02), and all standardized differences remained well below conventional thresholds for meaningful imbalance (|*h*| < 0.20). Detailed comparisons of the full sample and the analytical subsample are provided in the OSF repository (see below).

### Measures

#### Sport and exercise participation

Because the present study was conducted in Germany and the survey was administered in German, interpreting the findings requires consideration of the meaning of the German term Sport, which differs somewhat from the distinction between “sport,” “exercise,” and “physical activity” commonly used in English. In German usage, Sport refers broadly to deliberately pursued leisure-time physical activities for which individuals typically set aside dedicated time. Such activities often involve changing into sportswear and include hiking, gym training, football, yoga, cycling, dance, or skateboarding. The concept therefore extends beyond what would often be labelled exercise in English, because it includes not only fitness-oriented training but also recreational, social, experiential, and lifestyle-oriented forms of movement. At the same time, it does not refer to total physical activity in the public-health sense. Routine daily movement such as climbing stairs, gardening, active transport, or walking the dog would usually not be considered Sport in this sense. Throughout this article, we therefore use the combined term sport and exercise to capture this broader German concept on which our German-language survey was based [37, 38]. Accordingly, the present measure reflects sport and exercise participation rather than total physical activity or guideline-defined moderate-to-vigorous physical activity.

To capture sport and exercise engagement, we assessed (a) whether individuals currently participated in sport or exercise and (b) the amount of time invested among those who were active. Current participation was assessed with a yes/no question asking whether participants engaged in sport or exercise activities. Participants reporting being active also indicated the typical intensity of their activity (mostly strenuous enough to induce sweating versus mostly moderate without strong sweating).

To quantify engagement volume, respondents were asked to think about their activity behavior during the previous months (spring, summer, and autumn of the current year). Following the format used in recent population health surveys [39], they reported (a) how many days per week they usually engaged in sport or exercise during this period and (b) the approximate total number of minutes spent on sport or exercise per week.

#### Affective valence of physical education memory (PEMV)

PEMV was assessed with a retrospective item asking participants how they had experienced PE during their school years (“Did you like physical education at school?”). Response options ranged from very negative to very positive evaluations (“I found it terrible”, “not so good”, “mixed”, “good”, “very good”). PEMV was included as a numeric covariate, with response categories recoded from − 2 to + 2 (i.e., centered at 0).

#### Biographical timing of formative sport or exercise experiences (FSEE)

Participants indicated the life phase in which they identified the most formative experiences for their present relationship with sport and exercise. Response options covered six life phases: childhood, adolescence, higher education or vocational training, adult life following education or vocational training, retirement or older adulthood, and “don’t know”. For the analyses, the three adult phases were collapsed, and childhood, adolescence, adulthood, and “don’t know” were included as a four-level factor using treatment contrasts with childhood as the reference category.

### Sociodemographic variables

Participants reported sex (female, male), age in years, highest educational attainment (low, mid, high), and federal state of residence within Germany. ISCED levels (0–8) were collapsed into three categories: low (0–2), mid (3–4), and high (5–8). Effects of sex and education were tested using Helmert contrasts. Age was included as a numeric covariate centered at 47 years; for descriptive analyses and a post-hoc LMM, participants were additionally grouped into the four age strata defined in the sampling design (18–29, 30–44, 45–64, and 65–74 years).

### Statistical analysis and modeling

Our modeling strategy distinguished among three conceptually different aspects of adult sport and exercise participation: whether individuals currently participated at all (“gatekeeping”), the amount of engagement among active individuals (“amplification”), and the way engagement was organized across the week (“organization”).

Because weekly sport and exercise minutes showed a substantial proportion of zero values together with a strongly right-skewed distribution of positive values, gatekeeping was analyzed using a hurdle model with a zero-inflated gamma specification [40, 41]. This two-part approach separates (a) the probability of reporting no sport or exercise from (b) the number of weekly minutes among those who were active. The zero-component included PEMV, FSEE, their interaction, and sociodemographic covariates (age, sex, and education). The positive-minutes component modeled weekly minutes among active respondents; only sex improved model fit in this component.

To examine amplification among active adults only (i.e., respondents reporting at least one day per week of sport and exercise), we estimated a linear mixed model (LMM) for overall engagement volume. Volume was represented by a composite indicator (principal component; pc1), reflecting the standardized average of log-transformed weekly participation frequency and total weekly minutes of sport and exercise. The model included PEMV, FSEE, their interaction, age, sex, and education as fixed effects, with a random intercept for federal state to account for regional clustering.

To examine organization of participation style among active adults, we estimated a second LMM using a second indicator (pc2) representing the relative balance between more frequent shorter sessions and fewer longer sessions (i.e., the standardized difference between weekly participation frequency and total weekly minutes of sport and exercise). Positive values indicate relatively frequent, shorter sessions, whereas negative values indicate fewer but longer sessions. The model included PEMV, FSEE, age, sex, and education as fixed effects, again with a random intercept for federal state.

To further examine potential nonlinearity in age effects for participation style, an additional model replaced continuous age with the four age groups designed in the sampling design (18–29, 30–44, 45–64, and 65–74 years), represented by prespecified orthogonal contrasts reflecting theoretically meaningful life-course comparisons.

Across analyses, nested sets of theoretically motivated candidate models of increasing complexity were compared using likelihood ratio tests (LRTs) and information criteria (AIC/BIC). Model selection was based on improvements in fit, with a decrease of at least five AIC units indicating a meaningful improvement. Following parsimony principles (Bates et al., 2018), the most parsimonious model providing adequate fit is reported. Full model specifications and model comparison results are available in the OSF repository.

Results are reported on interpretable scales, including odds ratios for the gatekeeping component, multiplicative effects for weekly minutes among active respondents, and regression coefficients for the volume (pc1) and participation-style (pc2) models, together with 95% confidence intervals.

### Data availability and reproducibility

All analyses were conducted in R. Data, analysis scripts, full model specifications, model comparison results, and the complete results of all supplementary statistical analyses (including follow-up contrast tests) are available through the Open Science Framework (https://osf.io/s2gdq).

## Results

**Fig. 1:**
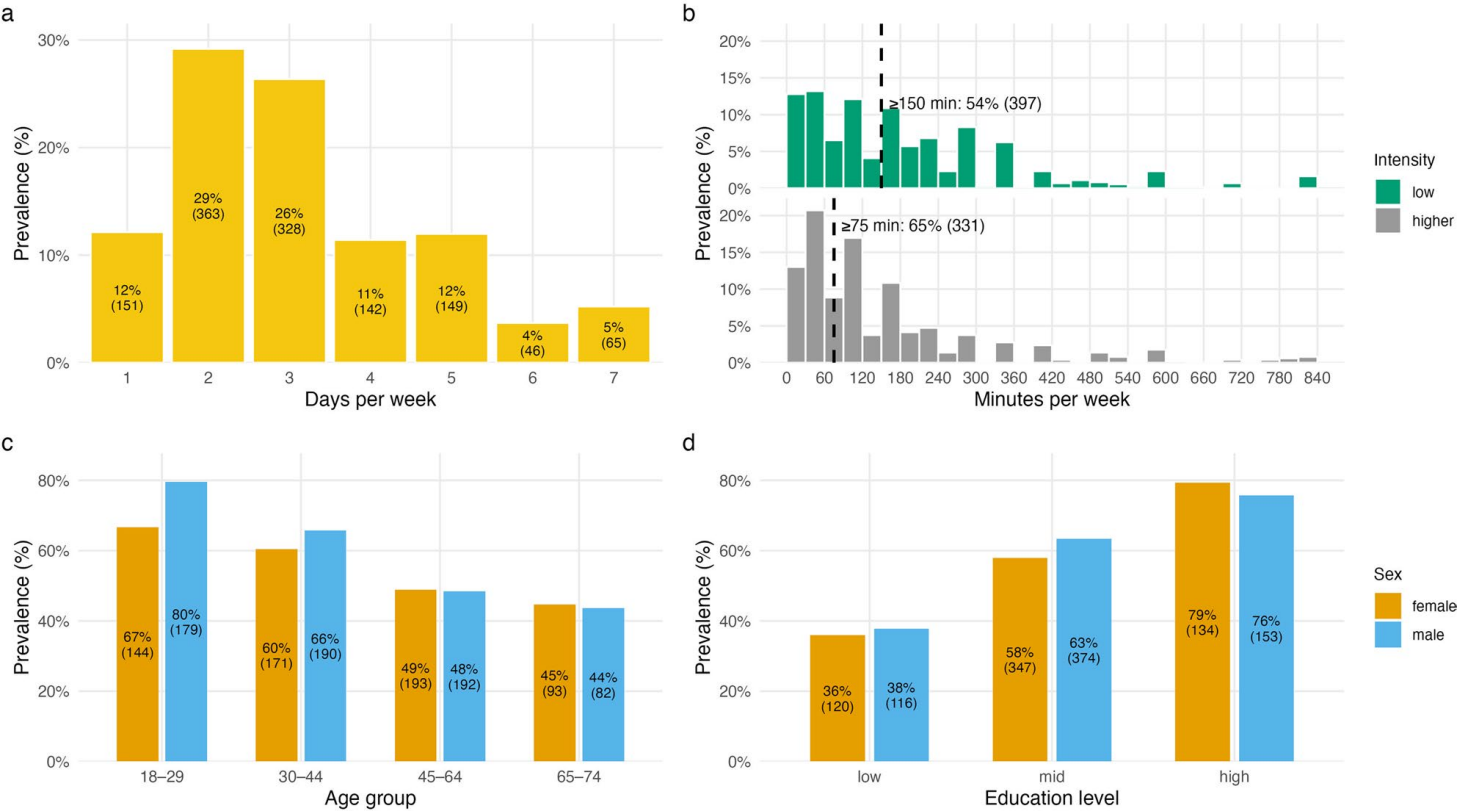
Sport and exercise participation in adulthood by intensity, age, sex, and education. **a** Distribution of weekly participation frequency among respondents reporting ≥ 1 day of sport or exercise (*n* = 1,244 of *N* = 2,201). Bars represent the prevalence of different participation frequencies; percentages are shown in the bars, and numbers in parentheses indicate the corresponding sample sizes (n). **b** Distribution of weekly minutes of sport or exercise among active participants, separated by typical activity intensity (lower-intensity vs. more physically demanding activities). Dashed vertical lines indicate commonly cited public-health reference thresholds (150 and 75 min per week) corresponding to guideline-relevant activity levels. Percentages and numbers in parentheses indicate the proportion and corresponding sample size (n) reaching these reference thresholds within each intensity category. **c** Prevalence of sport or exercise participation by age group and sex. Bars represent the proportion of respondents reporting ≥ 1 day of participation per week; percentages are shown in the bars, numbers in parentheses indicate the corresponding sample sizes (n). **d** Prevalence of participation by educational attainment and sex. Bars represent the proportion of respondents reporting ≥ 1 day of participation per week; percentages are shown in the bars, numbers in parentheses indicate the corresponding sample sizes (n)

Overall, 57% of respondents reported engaging regularly in sport and exercise. Among active respondents, participation occurred most often two to three days per week (56% combined), whereas engagement on five or more days was less frequent (21%) and once-weekly participation accounted for 12% (Fig. 1a).

Among those reporting predominantly lower-intensity activities, 54% accumulated at least 150 min per week; among those reporting higher-intensity activities, 65% accumulated at least 75 min per week (Fig. 1b). These proportions are broadly plausible in light of commonly cited public-health thresholds and population-level activity estimates for Germany [7, 42, 43]. However, these values should not be interpreted as direct indicators of guideline adherence, as the present measure captures dedicated sport and exercise participation rather than guideline-defined physical activity and does not separately assess muscle-strengthening activities [44].

Participation declined progressively across age groups (Fig. 1c). Engagement was highest in young adulthood and lowest in older adulthood, with marked sex differences in younger cohorts that narrowed substantially from midlife onward. Educational attainment showed a clear positive gradient (Fig. 1d), with participation increasing stepwise from the low-education group to the mid- and high-education groups. The overall direction and magnitude of these sociodemographic gradients are consistent with representative monitoring studies in Germany [7, 42, 43].

Among respondents who reported affective memories of school PE (analytical subsample, *n* = 1,917), formative sport and exercise experiences were most often remembered as occurring in childhood (41%), followed by adolescence (27%) and adulthood (26%), whereas 7% of respondents indicated that they did not know. Although early life stages predominated, a substantial proportion of respondents remembered decisive experiences later in life.

Retrospective evaluations of school PE covered the full response scale: 21% described their experiences as horrible, 13% as not so good, 28% as mixed, 18% as good, and 20% as very good. This distribution illustrates considerable heterogeneity in how school PE is remembered in adulthood.

### Biographical gatekeeping of sport and exercise engagement

Among participants who reported engaging in sport or exercise, men accumulated slightly more weekly minutes than women (PR = 1.08, 95% CI 1.03–1.14, *p* = 0.002), corresponding to roughly 8% more time invested (Table 2).

**Table 2:** Zero-inflated gamma hurdle model of weekly sport and exercise

Probability of reporting no sport or exercise (zero-inflation component)				
*Predictor*	*OR*	*95% CI*	*z*	*p*
PEMV: centered	0.61	[0.54, 0.68]	-8.87	< 0.001
FSEE2: adolescence vs. childhood	1.28	[1.00, 1.64]	1.99	0.047
FSEE3: adulthood vs. childhood	0.62	[0.48, 0.80]	-3.66	< 0.001
FSEE4: don’t know vs. childhood	2.17	[1.40, 3.36]	3.48	< 0.001
Sex: male vs. female	1.01	[0.91, 1.12]	0.24	0.809
Age: centered at 47 years	1.03	[1.02, 1.03]	7.70	< 0.001
Education1: mid vs. low	0.66	[0.59, 0.74]	-7.13	< 0.001
Education2: high vs. mean of mid and low	0.66	[0.60, 0.72]	-8.41	< 0.001
PEMV × FSEE2	1.12	[0.94, 1.34]	1.26	0.206
PEMV × FSEE3	1.90	[1.58, 2.30]	6.67	< 0.001
PEMV × FSEE4	1.50	[1.04, 2.17]	2.17	0.030

***Model dispersion***				
***Parameter***	***Estimate***	***95% CI***		
Gamma dispersion	0.85	[0.82, 0.88]		

**Positive minutes among active respondents (conditional gamma component)**				
***Predictor***	***PR***	***95% CI***	***z***	***p***
Sex: male vs. female	1.08	[1.03, 1.14]	3.03	0.002

*OR* odds ratio for reporting no sport or exercise (zero-inflation component), *PR* prevalence ratio, representing the multiplicative difference in weekly minutes among respondents reporting non-zero activity (conditional gamma component), *PEMV* physical education memory valence, *FSEE* formative sport and exercise experience (treatment contrasts). Education = Helmert contrasts. The model includes a gamma dispersion parameter (see estimate and 95% CI in table)

**Fig. 2:**
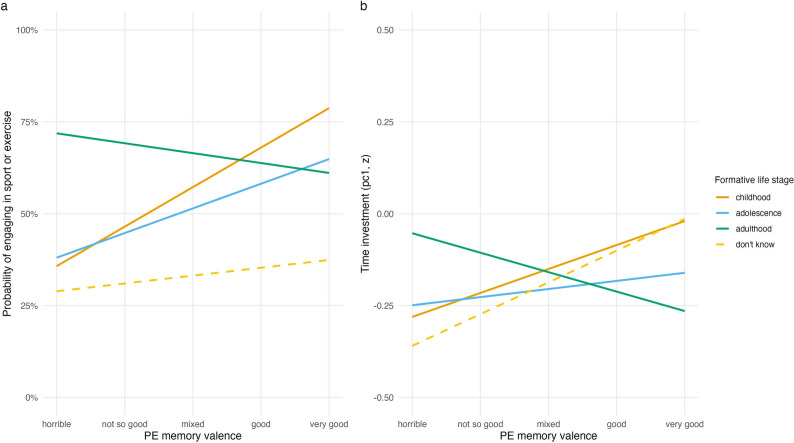
Biographical gatekeeping and time investment in sport and exercise. **a** Probability of engaging in sport or exercise (that is, reporting > 0 weekly minutes) as a function of valence of PE memory and the self-identified life stage of formative sport and exercise experiences (FSEE). Lines represent model-based linear trends estimated from the gatekeeping component of the hurdle model. Colors denote the reported formative life stage; the “don’t know” category is shown with a dashed line. **b** Relative time investment among active participants (that is, respondents reporting ≥ 1 day of sport or exercise), expressed as the first principal component of weekly activity volume (pc1; *z*-standardized), by valence of PE memory and FSEE. Lines represent model-based predictions from the linear mixed model of engagement volume among active adults. Higher values indicate greater overall time investment among those who are active

In the gatekeeping component, PEMV showed a robust association with engagement, but this association depended on the reported timing of formative sport and exercise experiences (FSEE × PEMV; Table 2; Fig. 2a). In the reference group of respondents who identified childhood as formative, higher PEMV was associated with substantially lower odds of reporting no sport or exercise (OR = 0.61, 95% CI 0.54–0.68, *p* < 0.001). The corresponding interaction indicated that this association did not differ significantly for respondents identifying adolescence as formative (interaction: OR = 1.12, *p* = 0.206), whereas it was significantly attenuated among those identifying adulthood as formative (interaction: OR = 1.90, *p* < 0.001). Follow-up simple slope analyses confirmed that PEMV remained negatively associated with non-engagement among respondents identifying childhood and adolescence as formative (both *p* < 0.001), but not among those identifying adulthood as formative (*p* = 0.067), suggesting a decoupling of PEMV from participation in this subgroup (see OSF).

Beyond these biographical effects, the probability of no sport or exercise increased with age (OR = 1.03 per year, 95% CI 1.02–1.03, *p* < 0.001) and showed a clear educational gradient, with higher education associated with lower odds of non-engagement (Table 2). Differences between FSEE categories were also evident at the average level of PEMV, indicating that biographical timing related to engagement over and above PEMV (Table 2).

### Biographical amplification of time investment

We next restricted the analyses to respondents reporting any sport or exercise (days ≥ 1). Among active adults, positive PEMV was associated with greater sport and exercise volume (pc1; β = 0.07, 95% CI 0.01–0.12, *p* = 0.027) (Table 3; Fig. 2b). However, this association differed by biographical timing: respondents who identified adulthood as their critical life phase showed a weaker association between PEMV and engagement volume than those identifying childhood (PEMV × FSEE[adulthood]: β = −0.12, 95% CI − 0.21 to − 0.03, *p* = 0.009; Fig. 2b). No corresponding moderation was evident for adolescence or “don’t know” relative to childhood (Table 3).

**Table 3:** LMM for total volume of sport and exercise among active adults (pc1)

Fixed effects				
*Predictor*	*β*	*95% CI*	*t*	*p*
(Intercept)	-0.00	[-0.09, 0.09]	-0.07	0.943
PEMV: centered	0.07	[0.01, 0.12]	2.22	0.027
FSEE2: adolescence vs. childhood	-0.05	[-0.19, 0.08]	-0.80	0.422
FSEE3: adulthood vs. childhood	-0.01	[-0.13, 0.12]	-0.13	0.893
FSEE4: don’t know vs. childhood	-0.04	[-0.30, 0.23]	-0.27	0.787
Sex: male vs. female	0.06	[0.01, 0.11]	2.30	0.021
Age: centered at 47 years	0.002	[-0.002, 0.005]	0.92	0.358
Education1: mid vs. low	0.04	[-0.03, 0.11]	1.09	0.277
Education2: high vs. mean of mid and low	0.05	[0.00, 0.09]	2.20	0.028
PEMV × FSEE2	-0.04	[-0.14, 0.05]	-0.89	0.375
PEMV × FSEE3	-0.12	[-0.21, -0.03]	-2.60	0.009
PEMV × FSEE4	0.02	[-0.21, 0.26]	0.18	0.860

**Random effects**				
***Component***	***SD***			
State (intercept)	0.03			
Residual	0.82			

The first principal component (pc1) represents total sport and exercise volume and is computed as the mean of z-standardized log(days/week) and z-standardized log(minutes/week). PEMV = physical education memory valence. FSEE = formative sport and exercise experience (treatment contrasts). Education = Helmert contrasts. The model includes random intercepts for state

Men exhibited slightly higher volume than women (β = 0.06, 95% CI 0.01–0.11, *p* = 0.021), and higher education was associated with greater volume. Age showed no clear association with volume in this active subsample (β = 0.002, 95% CI − 0.002 to 0.005, *p* = 0.358). Together, these results suggest that among active adults, PEMV was only modestly related to engagement volume, with the weakest association observed among respondents identifying adulthood as their formative period.

### Life phase organization of participation style

Finally, we examined whether active adults differ in how they organize sport and exercise across the week. In contrast to the preceding models, neither PEMV nor the timing of FSEE was associated with participation style (all p values ≥ 0.25) (Table 4). Sex differences were also small and not statistically significant (*p* = 0.193). Thus, there was no reliable evidence that temporal organization of sport and exercise among active adults depended on PEMV and FSEE.

**Table 4:** Linear mixed models predicting participation style (pc2)

Fixed effects								
*Models*	*Main LMM*				*Post-hoc LMM*			
*Predictor*	*β*	*95% CI*	*t*	*p*	*β*	*95% CI*	*t*	*p*
(Intercept)	-0.01	[-0.13, 0.11]	-0.18	0.860	-0.00	[-0.13, 0.12]	-0.05	0.964
PEMV: centered	-0.01	[-0.06, 0.04]	-0.46	0.642	-0.02	[-0.07, 0.04]	-0.59	0.556
FSEE2: adolescence vs. childhood	0.10	[-0.07, 0.26]	1.14	0.253	0.09	[-0.07, 0.26]	1.08	0.278
FSEE3: adulthood vs. childhood	0.05	[-0.11, 0.22]	0.62	0.538	0.04	[-0.13, 0.20]	0.44	0.659
FSEE4: don’t know vs. childhood	0.05	[-0.29, 0.40]	0.31	0.756	0.05	[-0.29, 0.39]	0.28	0.782
Sex: male vs. female	-0.05	[-0.11, 0.02]	-1.30	0.193	-0.04	[-0.11, 0.02]	-1.25	0.211
Age: centered at 47 years	-0.01	[-0.01, -0.00]	-3.12	0.002	–	–	–	–
Age1: 30–64 vs. [18–29; 65+] years	–	–	–	–	0.09	[-0.01, 0.18]	1.84	0.067
Age2: 18–19 vs. 65 + years	–	–	–	–	0.11	[-0.00, 0.22]	1.95	0.051
Age3: 30–44 vs. 45–64 years	–	–	–	–	0.10	[0.02, 0.19]	2.40	0.017
Education1: mid vs. low	-0.11	[-0.20, -0.02]	-2.47	0.014	-0.12	[-0.21, -0.03]	-2.53	0.012
Education2: high vs. mean of mid and low	-0.03	[-0.09, 0.02]	-1.23	0.219	-0.04	[-0.09, 0.02]	-1.29	0.198

**Random effects**								
***Component***	***SD***				***SD***			
State (intercept)	0.07				0.08			
Residual	1.09				1.09			

The second principal component (pc2) represents participation style and is computed as the difference between *z*-standardized log(days/week) and *z*-standardized log(minutes/week) (i.e., days minus minutes). Positive values indicate a relative emphasis on more frequent, shorter sessions; negative values indicate a relative emphasis on fewer, longer sessions. *PEMV* physical education memory valence, *FSEE* formative sport and exercise experience (treatment contrasts). Education = Helmert contrasts. Age contrasts in the post-hoc model represent orthogonal comparisons across four age groups. Both models include random intercepts for state

Instead, participation style varied systematically with age and education. Age showed a negative association with pc2 (β = −0.007, 95% CI − 0.011 to − 0.003, *p* = 0.002), indicating a gradual shift across adulthood from frequency-oriented engagement toward longer session durations. Participants with mid-level education also tended to exhibit a stronger duration-oriented participation style than those with low education (β = −0.11, 95% CI − 0.20 to − 0.02, *p* = 0.014).

**Fig. 3:**
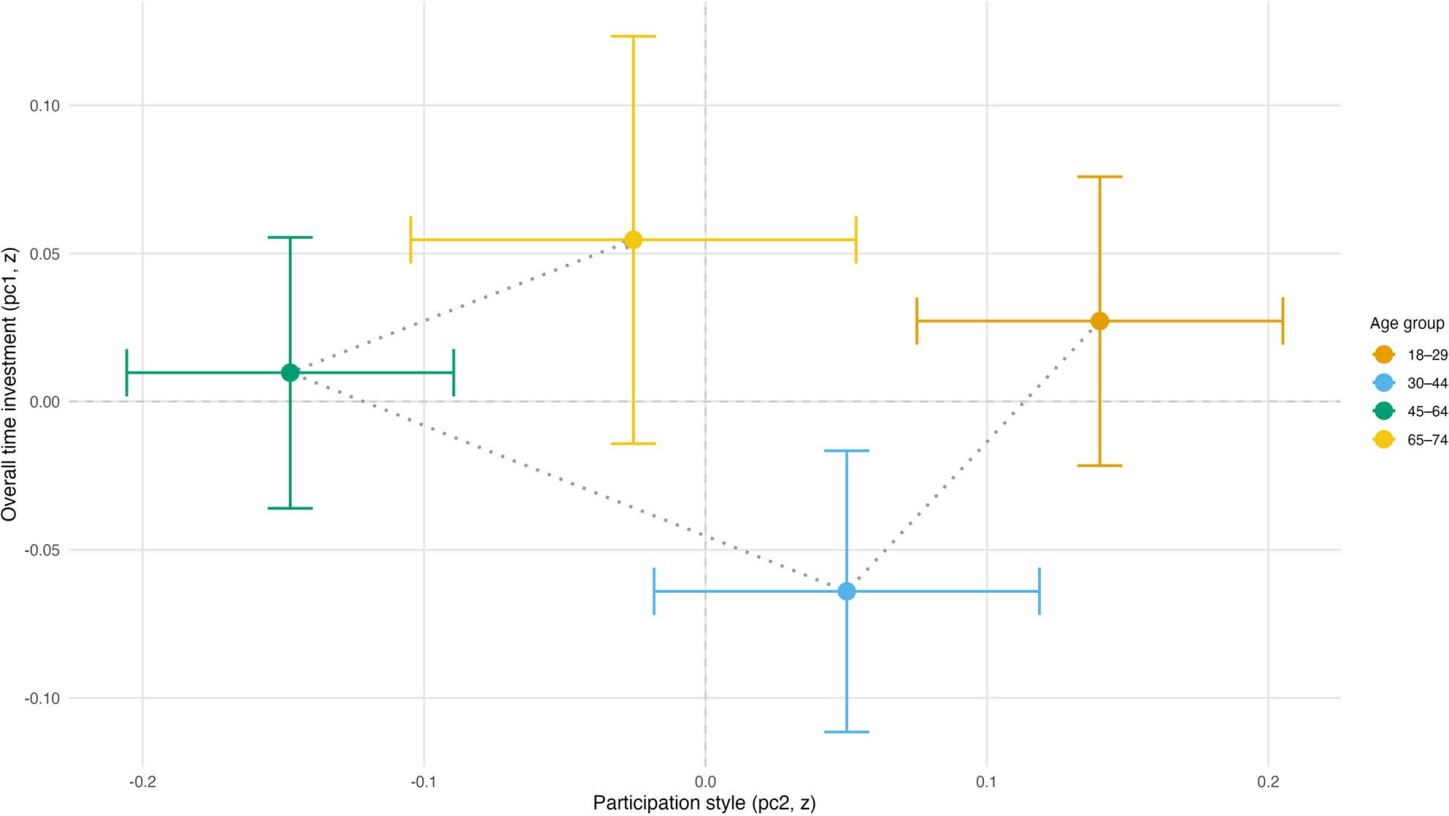
Participation style shifts from frequent to longer exercise sessions across adulthood. Mean participation style and overall time investment across four adult age groups (18–29 years, *n* = 276; 30–44 years, *n* = 305; 45–64 years, *n* = 338; 65–74 years, *n* = 160) among respondents reporting ≥ 1 day of sport or exercise (*n* = 1,079) within the analytical sample (*n* = 1,917). The horizontal axis shows participation style (pc2), defined as the difference between z-standardized log(days per week) and z-standardized log(minutes per week). Positive values indicate relatively frequent, shorter exercise sessions (frequency-oriented engagement), whereas negative values indicate fewer but longer sessions (duration-oriented engagement). The vertical axis shows overall time investment (pc1), the first principal component capturing combined variation in participation frequency and total weekly minutes. Points represent age-group means. Error bars denote ± s.e.m. in both dimensions. Labels next to points identify the corresponding age group. The dotted path links age groups in ascending order to aid visual interpretation of the pattern across adulthood

Additional analyses using contrasts between the predefined age-groups (young adulthood, 18–29; early midlife, 30–44; midlife 45–64; older adulthood, 65–74 years) suggested a differentiated pattern over the adult life course (Fig. 3; Table 4). Relative to young adulthood, midlife adults (45–64 years) showed a significantly stronger duration-oriented participation style (contrast: β = 0.10, 95% CI 0.02–0.19, *p* = 0.017), whereas contrasts involving early midlife (30–44 years) and older adulthood (65–74 years) were smaller and less precise. Descriptively, younger adults (18–29 years) tended to engage through relatively frequent, shorter sessions, whereas early midlife (30–44 years) was characterized by reduced overall engagement. From midlife onward (45–64 years), participation was characterized by fewer but longer sessions, accompanied by a recovery of overall activity volume. In older adulthood (65–74 years), both frequency and total time investment were comparatively high, resulting in the highest overall engagement and a more balanced participation pattern. To illustrate the practical magnitude of these differences, weekly participation frequency varied by roughly 1.4–1.7 days (s.d.), weekly minutes by about 150–180 min (s.d.), and average session durations by 36–46 min (s.d.). Overall, individual variability in participation was substantial.

Taken together, the three modeling steps indicate that different aspects of adult sport and exercise participation are associated with different sets of predictors. PEMV and the timing of FSEE were most strongly associated with whether respondents engaged in sport or exercise at all. Among those who were active, associations with overall time investment were comparatively small. By contrast, the temporal organization of participation showed no detectable relationship with PEMV or FSEE but varied systematically with age.

## Discussion

We investigated how the affective valence of adults’ memories of school physical education (PEMV), and the remembered timing of formative sport and exercise experiences (FSEE), relate to different dimensions of adult sport and exercise participation. The findings suggest that participation is not best understood as a single behavioral outcome, but as a layered structure consisting of three components: whether individuals engage in sport and exercise at all, how much time they invest once active, and how participation is organized within everyday life. Associations with PEMV were strongest for engagement versus non-engagement, weaker for the amount of activity among active individuals, and absent for the temporal organization of participation. In addition, the magnitude of these associations varied systematically according to the life-course timing of FSEE. Together, these findings suggest that remembered experiences from childhood and adolescence are most strongly linked to whether sport and exercise become part of adult behavioral repertoires at all, whereas the organization of participation in everyday life appears to be less closely related to remembered experiences and more closely tied to current life circumstances.

The clearest pattern concerned the distinction between engagement and non-engagement. Adults who remembered their school PE experiences more positively were substantially more likely to report participating in sport and exercise, whereas those with more negative PEMV were more likely to report no engagement at all. This pattern suggests that remembered PE experiences are closely linked to whether sport and exercise become part of adult behavioral repertoires. To the extent that these memories reflect earlier experiences, they may indicate a form of motivational gatekeeping. Such experiences would not be expected to determine behavior directly but rather to influence how individuals evaluate later opportunities for movement [45]. When PE is remembered as enjoyable, competence-supportive, or socially meaningful, later opportunities for engagement may appear inviting; when PE is remembered more negatively, the same opportunities may be interpreted as less attractive or less personally relevant. In this sense, remembered PE experiences may form part of the reference framework through which individuals decide whether sport and exercise belong in their lives at all [46, 47].

The apparent motivational gatekeeping associated with PEMV was largely confined to respondents identifying childhood or adolescence as their formative period. Among the smaller subgroup identifying adulthood as formative (26%), this association was no longer evident. Within the childhood and adolescence groups, remembered PE and adult engagement were closely aligned: individuals with more positive PEMV were more likely to be active, whereas those recalling PE more negatively were more likely to report non-engagement. This pattern is consistent with the possibility that, when formative experiences are located early in life, the affective tone of PE memories remains closely linked to later orientations toward sport and exercise [48].

This apparent decoupling becomes more interpretable from a life-course perspective. Individuals who develop a meaningful engagement with sport and exercise only in adulthood may do so in contexts that differ substantially from school PE [49, 50]. Contemporary adults have access to a much broader range of activities and participation settings than those typically encountered during childhood or adolescence, allowing some individuals to discover forms of movement that better match their preferences, interests, or life circumstances. From this perspective, later formative experiences may represent turning points [32] that open motivational pathways not established earlier in life.

One possible interpretation of this pattern is that some individuals encounter what might be described as “second entry points” into sport and exercise. However, because the present study did not assess the specific experiences or mechanisms underlying adult-formative trajectories, this interpretation remains tentative. Future longitudinal research will be needed to determine whether later formative experiences indeed reflect opportunities to discover new forms of movement and thereby weaken the association between negative PE memories and adult participation.

Beyond these life-course patterns, the findings indicate that different dimensions of sport and exercise participation follow different explanatory logics. Remembered PE experiences were most strongly associated with whether individuals engaged in sport and exercise at all, whereas their association with activity volume among active adults was comparatively modest. This suggests that once engagement is established, other factors such as available time, personal goals, or everyday constraints may play a larger role in shaping how much activity individuals undertake [31, 51].

An even clearer distinction emerged for participation style. Whether individuals accumulated activity through frequent shorter sessions or fewer longer bouts was unrelated to PE memories or formative sport timing but varied systematically with age. This suggests that the temporal organization of participation may be more closely tied to the demands and rhythms of everyday life across adulthood than to earlier movement experiences [52, 53].

Placed in the context of existing research, these findings extend a still emerging line of work on remembered PE experiences. Earlier retrospective studies have linked remembered enjoyment in PE to later physical activity behavior and identified specific negative experiences, such as embarrassment or social exclusion, as potential sources of unfavorable attitudes toward movement [12–14]. However, these studies typically treated participation as a single outcome. By distinguishing between engagement versus non-engagement, activity volume, and participation style, the present study suggests that the motivational relevance of PE memories is concentrated primarily at the point where individuals decide whether sport and exercise become part of their lives at all.

At the same time, these findings should be interpreted with potential cohort effects in mind. Participants experienced school PE across several decades during which educational practices and opportunities for sport and exercise changed substantially. In Germany, this period also spans the reunification of the country in 1990, meaning that some participants attended school in the former German Democratic Republic, whereas others experienced PE in the Federal Republic of Germany or the reunified education system [54–56]. Consequently, remembered PE experiences may reflect not only individual biographies but also historical differences in the contexts in which these experiences occurred. Future studies should examine more explicitly how developmental and cohort effects jointly contribute to remembered PE experiences and their associations with adult behavior.

Beyond such cohort-related considerations, interpreting these associations also requires acknowledging the reconstructive nature of autobiographical memory [23–26, 57]. Retrospective accounts of earlier sport and exercise experiences are inevitably shaped by later perspectives and reinterpretations, including individuals’ current engagement in sport and exercise. Consequently, current sport and exercise participation may not only reflect remembered PE experiences but may also influence how those experiences are reconstructed in adulthood. The present data therefore do not provide direct evidence that such experiences caused later behavior. Rather, the study captures adults’ current representations of these earlier experiences. However, the reconstructive nature of autobiographical memory does not render remembered experiences irrelevant. The ways in which individuals currently remember and narrate earlier experiences form part of the psychological framework through which they interpret their relationship with sport and exercise. Even if memories are filtered through later life narratives, they are unlikely to be arbitrary constructions and can reasonably be assumed to originate in experiences that were affectively meaningful at the time.

Taken together, the findings suggest that affectively colored movement experiences are most strongly associated with the threshold between engagement and disengagement. Earlier experiences may contribute to whether sport and exercise are perceived as attractive and personally relevant behavioral options at all. At the same time, motivational trajectories remain open across the life course. The presence of adults who report being active despite negative PE memories is consistent with the possibility that later experiences can create new entry points into engagement [45, 58]. Once individuals are already active, however, the amount and organization of activity appear to depend less on earlier affective experiences and more on the practical constraints and opportunities of everyday life [31, 52].

These findings also suggest practical implications for physical activity promotion and public health. Interventions may benefit from addressing not only current barriers to activity but also the affective meanings individuals attach to movement. Although such approaches remain largely exploratory, supporting individuals in identifying personally meaningful and positive forms of movement may complement more traditional behavior-change strategies [59].

Participation style may warrant particular attention from a public health perspective. Current physical-activity guidelines primarily emphasize total activity volume while allowing considerable flexibility in how activity is accumulated across the week [60]. The present findings suggest that adults make use of this flexibility differently across life phases. For example, younger adults in our sample tended to accumulate activity through relatively frequent, shorter sessions, whereas participation in midlife was characterized by fewer but longer sessions. In older adulthood, both participation frequency and overall time investment were comparatively high, resulting in the highest overall engagement and a more balanced participation pattern. These findings potentially reflect changing work demands, family responsibilities, retirement, and other life-course transitions. From a public-health perspective, providing life-phase-specific examples of how recommended activity levels can be achieved through different participation patterns may help individuals translate general recommendations into everyday practice.

The present findings also have implications for the design and delivery of school PE. Beyond delivering technically sound instruction or well-designed curricula, teachers may benefit from paying greater attention to how individual students actually experience PE lessons and what kinds of memories these experiences may leave behind. Supportive learning environments, inclusive teaching practices, and opportunities to experience competence, enjoyment, autonomy, and personal relevance may increase the likelihood that PE becomes remembered as a positive rather than aversive context. If such affective memories indeed contribute to later engagement, teacher education may benefit from placing greater emphasis not only on what is taught, but also on how PE is experienced by students.

Several limitations should be considered when interpreting the present findings. First, the analyses are based on cross-sectional survey data and retrospective reports, precluding causal conclusions. Current sport and exercise participation may itself shape how earlier PE experiences are reconstructed, and reciprocal influences cannot be disentangled within the present design. Second, PEMV was assessed using a single retrospective item. This approach provides only a broad summary evaluation and cannot disentangle potentially distinct aspects of remembered PE experiences, including enjoyment, perceived competence, teachers, peer interactions, or other features that may contribute to how PE is remembered. Third, although the study draws on a large population-based sample, the findings reflect the institutional and cultural context of PE and sport in Germany. While the underlying psychological mechanisms linking remembered affective experiences with later behavior are unlikely to be unique to one educational system, differences in PE curricula, institutional structures, and cultural traditions may influence the strength of these associations. Future cross-national research should therefore examine the generalizability of the present findings across educational contexts. Fourth, the analyses focused specifically on sport and exercise participation rather than total physical activity, and the observed patterns may not necessarily extend to other forms of everyday movement.

In conclusion, the present findings suggest that remembered movement experiences may have implications extending far beyond short-term physical outcomes because they can form part of the motivational architecture through which adults interpret sport and exercise. These findings therefore concern current representations of earlier experiences, which are inevitably reconstructed from the perspective of the present, rather than the objective reality of those experiences decades ago, although such representations can reasonably be assumed to originate in experiences that were meaningful at the time. School PE is one of the few contexts in which nearly all individuals encounter structured movement during formative years, making it a potential population-level gatekeeper for later engagement. When remembered in more positive terms, early movement experiences may contribute to motivational orientations that make sport and exercise feel like attractive and personally relevant aspects of adult life [61].

## Data Availability

The datasets analyzed during the current study, along with the analysis code and study materials, are openly available in the Open Science Framework (OSF) repository at https://osf.io/s2gdq.
